# Diffuse large B-cell lymphoma involving peripheral nervous system with IgM antibodies against GM1 and GD1b: A case report

**DOI:** 10.1097/MD.0000000000015049

**Published:** 2019-04-12

**Authors:** Zhaocai Jiang, Weina Ju, Shen Luo, Yu Yang

**Affiliations:** Department of Neurology and Neuroscience Center, First Hospital of Jilin University, Changchun, Jilin, China.

**Keywords:** autoimmunity, B-cell lymphoma, glycolipid GD1b, glycolipid GM1, peripheral neuropathy

## Abstract

**Rationale:**

The occurrence of peripheral neuropathy associated with non-Hodgkin's lymphoma (NHL) is uncommon. And autoimmunity may play an important role. We report a case of the patient with NHL, has sensorimotor demyelinating polyneuropathy.

**Patient concerns:**

The patient presented with a 1-month history of progressive numbness at the distal extremities and motor weakness of the lower limbs. Meanwhile, patient also endorsed a painful lump on her right cheek. And then the enlarged cervical and supra clavicular lymph nodes were observed on admission. Biopsy of the lymph nodes showed NHL. Serum IgM antibodies against GM1 and GD1b were also positive.

**Diagnosis:**

Biopsy of the lymph nodes showed NHL. Serum IgM antibodies against GM1 and GD1b were also positive. Thus, the patience was diagnosed with lymphoma and sensorimotor polyneuropathy.

**Interventions:**

Patient refused the further treatment.

**Outcomes:**

After 11-month follow-up, the weakness of bilateral lower limbs worsens.

**Lessons:**

We have presented a case of NHL involving peripheral polyneuropathy with IgM antibodies against GM1 and GD1b. Patients may initially present with peripheral nerve complications or develop them during the course of lymphoma, even when in remission. This could complicate the diagnosis of peripheral polyneuropathy secondary to NHL.

## 1 Introduction

Peripheral nervous system abnormalities occur in only 5% of patients with lymphoma.^[1]^ There have been isolated reports of patients with B-cell non-Hodgkin's lymphoma (NHL) involving polyneuropathy by autoantibodies against peripheral nerve glycolipid antigens. However, the exact mechanism has not been clarified, autoimmunity may play an important role.^[2]^ Patients may initially present with peripheral nerve complications or develop them during the course of lymphoma, even when in remission. It is easy to cause misdiagnosis or missed diagnosis.^[3,4]^ We herein report a patient with initial symptom of peripheral neuropathy involved by NHL with IgM antibodies against GM1 and GD1b. The possible mechanism is also discussed in this paper.

## 2 Case report

The patient presented with a 1-month history of progressive numbness at the distal extremities and motor weakness of the lower limbs. Notably, she had lost 4 kg over the past half year. On admission, neurological examination showed painful paresthesia with a stocking-glove distribution, with strength of grade 4/5 in extremities. Additionally, tendon hyporeflexia was noted in the lower limbs. Laboratory results included the count of red blood cell, white blood cell, and the platelet within the normal range, serum antibodies to different virus (include herpes simplex virus, rubella virus, Epstein–Barr virus and cytomegalovirus) negative. Electroneurography documented absent sensory action potentials in the lower limbs (Table 1). Cerebrospinal fluid examination (CSF) showed a slight increase in protein concentration (46 mg/dL, normal range, 15–45 mg/dL). The cell count was 1×10^6^ cells/L (normal range, 0–8 cells/L) and without any abnormal cells. An enzyme-linked immunosorbent assay (ELISA) revealed the presence of IgM antibodies against GM1 and GD1b in the serum. Other paraneoplastic anti-neuronal antibodies, including anti-Hu, anti-Yo, anti-Ri, anti-CV2, anti-Tr, and anti-Ma2, were negative in both serum and CSF. Magnetic resonance imaging (MRI) of the cervical, thoracic, and lumbar spine showed degenerative changes.

**Table 1:**
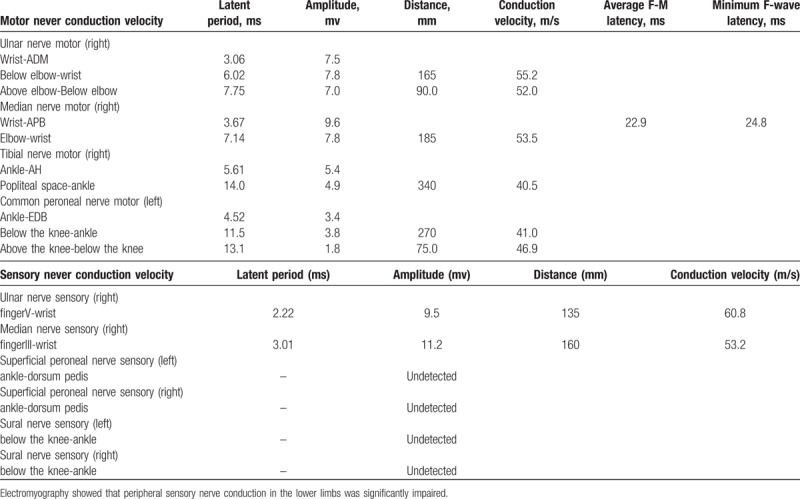
Electromyography.

**Figure 1:**
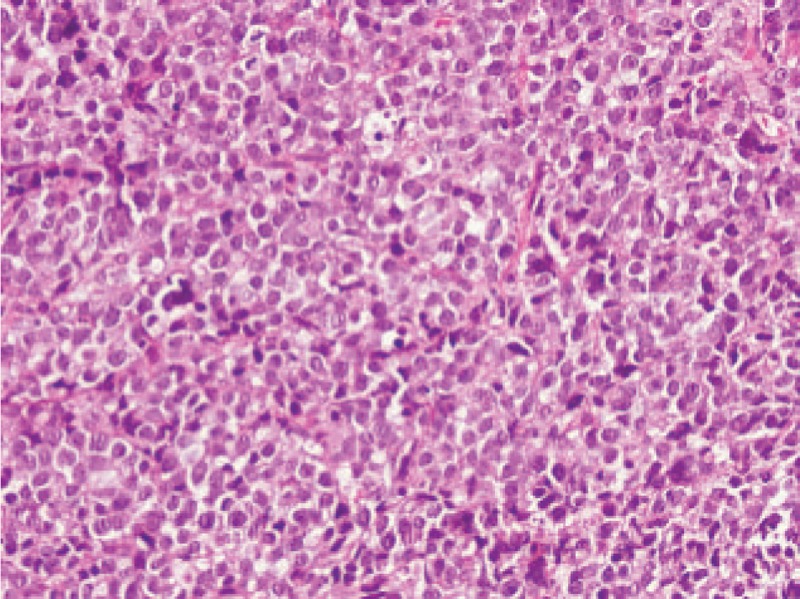
Biopsy of right cervical lymph node: Non-Hodgkin's malignant lymphoma, WHO classification: diffuse large B-cell lymphoma (HE staining, 400 x). HE = hematoxylin and eosin, WHO = World Health Organization.

The patient also had a painful lump on her right cheek for the past 2-month. Then the 3-dimensional CT of the paranasal sinus was performed in other hospital and demonstrated: the bone of the right maxillary sinus wall and sphenoid wing were destructed. No further workup was pursued until she was admitted to our hospital. Patient felt the pain on her cheek was worsening. An ultrasonography of systemic lymph nodes showed enlarged cervical and supra clavicular lymph nodes. Biopsy of the lymph nodes showed NHL of diffuse large B-cell type (Fig. 1). The results of immunohistochemical examination were as follows: Ki-67 (+90%), CD20 (+), PAX-5 (+),CD79α (+), CD3 (−), CD43 (partially +), Bcl-2 (+90%), CD5 (−), CD10 (+), Bcl-6 (+), MUM-1 (+), CD21 (−), c-Myc (+40%), Cyclin D1 (−), CD30 (−), CD23 (−), P53 (+<50%).^[5]^ Molecular pathologic findings indicated that DNA encoding the B-cell was rearranged. Based on the clinical features and auxiliary examination, the patient was diagnosed with lymphoma. The patient was referred to hematology/oncology for further treatment. After 11-month follow-up, the weakness of bilateral lower limbs worsens. Patient cannot walk by herself.

## 3 Discussion

The incidence of peripheral neuropathy in NHL is slightly higher than Hodgkin's lymphoma (HL). These are much more likely to occur in B-cell derived NHL, especially in advanced stage.^[3]^ These peripheral neuropathies occur most frequently by direct infiltration of nerve system, and maybe also secondary to immune system disturbance, infectious, metabolic disorder, directly compressing, radiotherapy, or chemotherapy.^[3,4,6–9]^ Biopsy is usually used to make a definite diagnosis of lymphomatosis caused by direct infiltration. Baehring and his colleagues^[10]^ proposed that the diffuse enlargement of nerves or roots suggests neoplastic infiltration in the proper clinical setting. This criteria allows a presumptive diagnosis without resorting to pathological proof by using the advances in neuroimaging. In this case, MRI of the cervical, thoracic, and lumbar spine did not show obvious abnormality. In addition, the patient did not experience infection, metabolic imbalances, radiation, and chemotherapy treatment. Examination of CSF revealed no abnormal tumor cells. Therefore, autoimmune-mediated peripheral neuropathy was highly suspected. Serum IgM antibody against GM1 and GD1b also support the assumption above. The first case of autoantibody-mediated neuropathy with gangliosides was reported in 1985,^[11]^ since then the characteristic of gangliosides has been well studied in the numerous literature. Gangliosides are found in all vertebrate plasma membranes with particular abundance in nerve cell membrane. GM1 and GD1b are 2 major kinds of gangliosides. GM1 is mainly distributed on the axonal membrane of motor nerve, especially at Ranvier nodules. The combination between GM1 and anti-GM1 antibody triggers the complement complex activation, which subsequently attacks the axonal membrane and demyelinates the peripheral motor nerve. Meanwhile, abundant of GD1b are also found in the large neurons of human dorsal root ganglia, sympathetic ganglia, as well as the paranodal myelin of motor and peripheral sensory nerves.^[12]^ The loss of GD1b may cause the abnormality of motor and sensation, and sensory ataxia.^[13–15]^ Interestingly, some autoantibodies show affinity for the terminal Gal (β1–3) GalNAc residue, which is shared by GM1 and GD1b^[16]^ (Table 2). Combination between those antibodies and the common terminal will cause multifocal sensorimotor neuropathy.^[17]^ The patient presented with abnormality of motor and sensation. As the disease progresses, the result of electroneurography may have a change if it was tested again. Shihashi et al^[8]^ summarized 6 cases of malignant lymphoma involving neuropathy with anti-disialosyl IgM antibodies in 2015 and 3 cases were diffuse large B-cell type. One case had IgM antibodies against GM1 and GQ1b in the serum. The second case had IgM antibodies against GD1b, GD3, GM1 and IgG antibodies against GalNAcGQ1a in the serum. The last case had IgM antibodies against GD3, GD2, GD1b, GT1b, GQ1b, GT1a and fucosyl GD1b. It is reasonable to suspect that IgM antibodies against GM1 and GD1b play an important role in diffuse large B cell lymphoma involving autoimmune peripheral neuropathy.

**Table 2:**
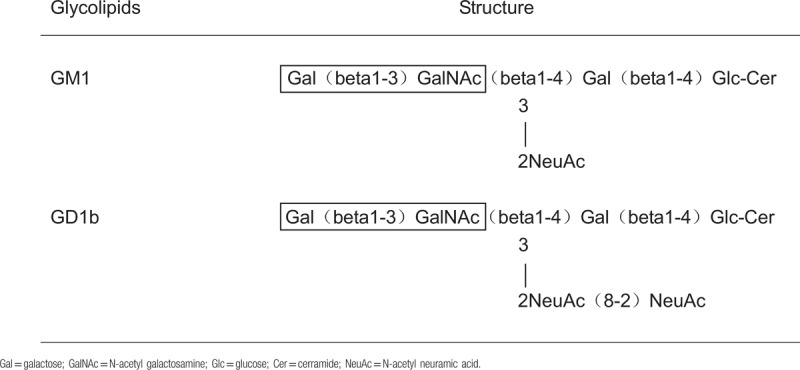
Structure of glycolipids, GM1 and GD1b.

**Figure 2:**
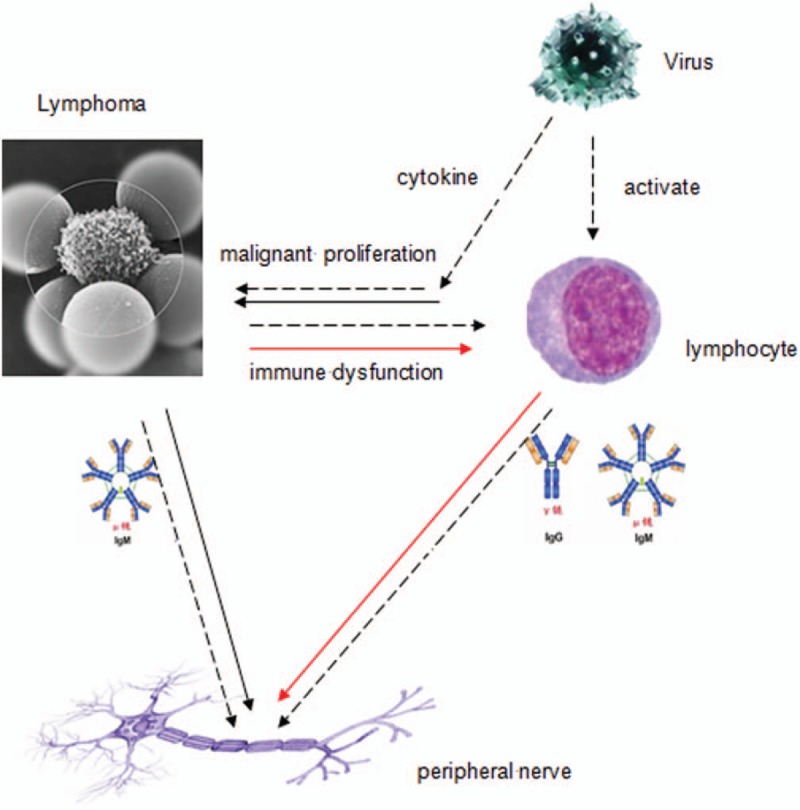
Immunologic mechanism of peripheral neuropathy caused by NHL (Mechanism 1: antibody produced by autoreactive B cells after malignant transformation; Mechanism 2: antibody produced by normal lymphocyte; Mechanism 3: viral infection). Informed written consent was obtained from the patient for publication of this case report and accompanying images. NHL = non-Hodgkin's lymphoma.

The immune-mechanisms of lymphoma-associated peripheral neuropathies still remain unknown, but several hypotheses have been proposed (Fig. 2). First, monoclonal autoantibodies (usually IgM paraprotein) produced by malignant transformation of the autoreactive B-cells, have the characteristics of natural autoantibodies^[1,18]^ which directed against self and non-self antigens without antigenic stimulation of secretory CD5+ B-cells.^[19]^ A patient was reported with a B-cell lymphoma producing autoantibody against peripheral nerve myelin glycolipids GM1 and GD1b. Immunofluorescent flow cytometry and thin-layer chromatographic immunostaining expressed that most of anti-GM1 and anti-GD1b antibody in the serum were monoclonal IgM of k type. ELISA demonstrated that this antibody was much more abundant in culture supernatant of lymphoma cell than that of normal lymphocyte.^[16]^ We suspect that the monoclonal IgM k autoantibody could be produced by the lymphoma cells. Second, autoantibodies may be secreted by non-lymphoma cells because of lymphoma-induced immune irregularity.^[2]^ As we all know, a single tumor clone can only secrete 1 kind of immunoglobulins. The detection of serum autoantibodies in some patients with NHL could be against various, yet distinct, antigens. This implies that these autoantibodies are not produced by malignant lymphoma cells.^[20]^ Lastly, virus infection could be the pathogenesis of both lymphoma and peripheral neuropathies. A process of viral antigen-specific stimulation can lead to clonal expansion and B-cell activation which may promote both lymphoma genesis and autoimmunity for peripheral nervous system.^[21]^ Futher examination, such as immunofluorescent flow cytometry or thin-layer chromatographic immunostaining, were not pursued to see if the antibodies against GM1 and GQ1b were monoclonal.

## 4 Conclusion

In general, the advanced tumors with higher stages intend to have a poorer prognosis and they are more likely to affect the nervous system.^[8]^ Based on other reports of lymphoma with immune-mediated neuropathy caused by anti-disialosyl ganglioside IgM antibodies, we propose that the early diagnosis and treatment of both peripheral neuropathy and NHL may improve prognosis of patients. Therefore, it is generally considered that the treatments should focus on tumor treatment and immunomodulation, mainly through intravenous immunoglobulin or plasma exchange.^[8,15]^ For patients with peripheral neuropathy of unknown etiology, we should be aware of the possibility of the underlying malignancy.

## Author contributions

**Conceptualization:** Zhaocai Jiang, Weina Ju, Yu Yang.

**Data curation:** Zhaocai Jiang, Weina Ju, Shen Luo.

**Investigation:** Zhaocai Jiang, Weina Ju, Shen Luo.

**Software:** Zhaocai Jiang, Shen Luo.

**Writing – original draft:** Zhaocai Jiang, Weina Ju.

**Writing – review & editing:** Yu Yang.
